# Elongated flap combined with pulley-traction method for the treatment of early duodenal bulb adenocarcinoma

**DOI:** 10.1055/a-2819-8381

**Published:** 2026-03-09

**Authors:** Qiuyu Wang, Rui Ji

**Affiliations:** 1 91623 Department of Gastroenterology, Qilu Hospital of Shandong University, Jinan, China

**Fig. 1:**
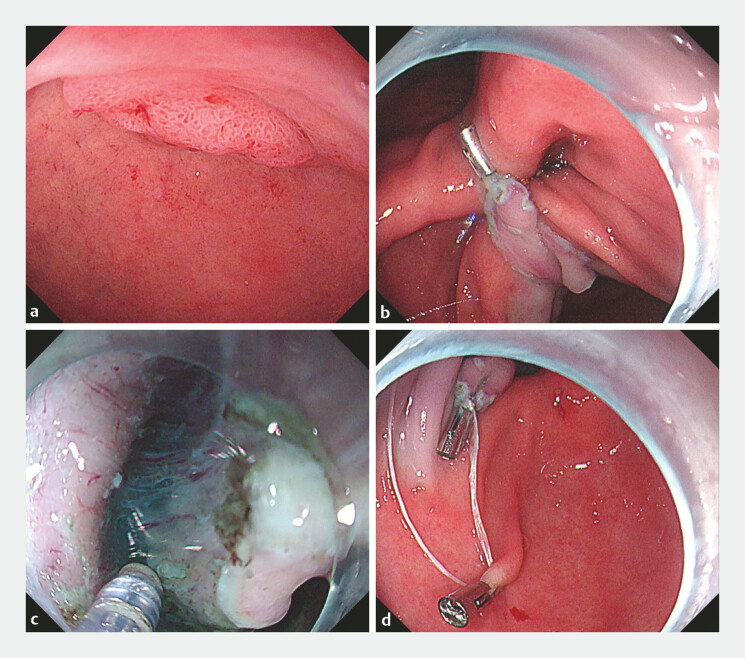
Endoscopic submucosal dissection. **a** A lesion on the lesser curvature of the duodenal bulb. **b** The first clip was placed on the edge of the elongated flap. **c** Clear visualization after external traction. **d** The second clip was placed on the contralateral gastric wall.

**Fig. 2:**
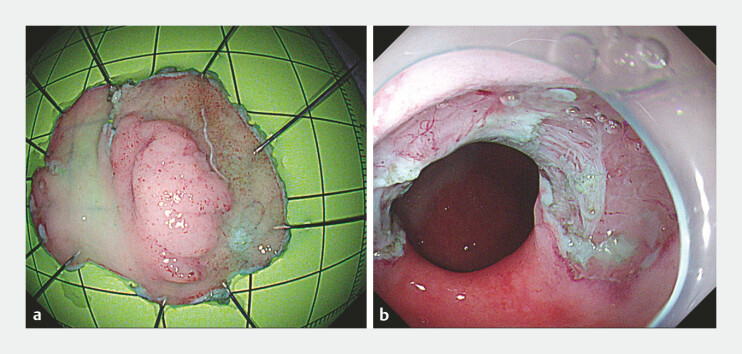
After dissection. **a** The specimen. **b** The mucosal defect on the antral side of the pylorus.

A 66-year-old man presenting with a loss of appetite was referred to our hospital due to a bulging lesion on the lesser curvature of the duodenal bulb. Endoscopy revealed a Paris 0–IIa lesion with a nodular elevation, measuring approximately 20 × 12 mm ( Fig. 1 **a** ). The procedure began with a linear mucosal incision at the anal side of the lesion, followed by a U-shaped coagulation marking on the normal gastric mucosa of the pyloric antrum. After the marking, the mucosa was incised and the submucosal layer was dissected toward the anal direction, thereby creating an elongated flap. To optimize exposure, a clip attached to dental floss was anchored to the edge of this flap ( Fig. 1 **b** ). External traction on the dental floss then supplied a constant pulling force, maintaining a clear submucosal view ( Fig. 1 **c** ). When further dissection became challenging, a second clip was placed on the contralateral gastric wall to alter the direction of traction ( Fig. 1 **d** ). This sequential, multi-directional setup provided stable and adaptable exposure, enabling complete and efficient en bloc resection of the lesion ( Fig. 2 **a** , Video 1 ). The total procedure time was 55 minutes without adverse events ( Fig. 2 **b** ). Histopathological examination confirmed high-grade intraepithelial neoplasia with focal intramucosal well-differentiated adenocarcinoma. All resection margins were free, and there was no lymphovascular invasion. Follow-up endoscopy at 2 months showed good mucosal healing, and biopsies revealed no evidence of tumor recurrence.

Endoscopic resection of early adenocarcinoma on the lesser curvature of the duodenal bulb.Video 1

In conclusion, this method effectively resolves the problem of difficult access in this challenging location. It may thus serve as a practical and effective option for managing similar early-stage neoplasms in challenging anatomical locations.

Endoscopy_UCTN_Code_TTT_1AO_2AG_3AD

